# Building Engagement Using Financial Incentives for Colorectal Cancer Screening (BENEFIT‐C) in a Rural Louisiana Federally Qualified Health Center

**DOI:** 10.1111/1475-6773.70011

**Published:** 2025-07-22

**Authors:** Laura M. Perry, Erin Peacock, Angela LeBlanc, LaKeisha Williams, Gary Wiltz, Marie Krousel‐Wood

**Affiliations:** ^1^ Center for Health Outcomes, Implementation, and Community‐Engaged Science (CHOICES) Tulane University School of Medicine New Orleans Louisiana USA; ^2^ John W. Deming Department of Medicine Tulane University School of Medicine New Orleans Louisiana USA; ^3^ Teche Health Franklin Louisiana USA; ^4^ Division of Clinical and Administrative Sciences College of Pharmacy, Xavier University of Louisiana New Orleans Louisiana USA; ^5^ Department of Epidemiology Tulane University Celia Scott Weatherhead School of Public Health and Tropical Medicine New Orleans Louisiana USA

## Abstract

**Objective:**

To evaluate the preliminary effectiveness and feasibility of a community‐engaged financial incentive intervention for increasing colorectal cancer (CRC) screening in a rural, low‐resource primary care setting.

**Study Setting and Design:**

A feasibility pilot trial at a federally qualified health center in the Acadiana region, Louisiana. Participants were identified via electronic health records (EHR), recruited by telephone, and randomized to intervention (*n* = 25) or control (*n* = 25). Both groups received incentives for flu and COVID‐19 vaccines. The intervention group also received incentives for CRC screening. Fisher's Exact tests evaluated between‐group differences in completion of CRC screening (primary outcome), flu, and COVID‐19 vaccines 2 months post‐enrollment.

**Data Sources and Analytic Sample:**

Eligible patients were aged 45–75, due for CRC screening, and English‐speaking. From November 11, 2023 to March 31, 2024, completion of each outcome was obtained from the EHR.

**Principal Findings:**

57% of interested individuals were eligible; 94% of eligible individuals enrolled. Participants were 70% female, 42% Black, 24% uninsured, with a mean age of 52.4 years. Completion of CRC screening was higher in the intervention versus control group (17 [68%] vs. 8 [32%], *p* = 0.02).

**Conclusion:**

Financial incentives significantly increased completion of CRC screening. Future research should confirm findings in larger samples and evaluate cost‐effectiveness to inform health system policies.

**Summary**

- What is known on this topic?
  ○ Louisiana has high rates of colorectal cancer (CRC) incidence and mortality, particularly in a rural region of southern Louisiana known as Acadiana.
  ○ Despite the demonstrated effectiveness of CRC screening for early detection and treatment of CRC, adherence rates are suboptimal in Louisiana federally qualified health centers and across the US.
  ○ Patient financial incentives can improve completion rates of CRC screening when combined with patient outreach and navigation, but evidence is limited when implemented in low‐income and under‐resourced primary care settings.
- What this study adds?
  ○ Participants randomized to receive outreach and a $50 financial incentive for CRC screening were more likely to complete a screening compared to a control group with no financial incentive for CRC screening.
  ○ Financial incentives may be effective and feasible to implement during routine outreach for CRC screening in primary care settings serving low‐income residents.

## 1 Introduction

Colorectal cancer (CRC) poses a burden in the US state of Louisiana, and more research is needed to identify strategies to reduce CRC‐related morbidity and mortality. CRC is the third most common cancer type in the US [1], and Louisiana has the fourth highest CRC incidence rate and the fifth highest CRC death rate statewide [1]. Moreover, the incidence of CRC is higher in Acadiana, Louisiana, a region known for its historically French and Cajun heritage [2, 3]. This could be due to multiple factors, including the rurality of Acadiana, shared environmental factors such as diet, or genetic risk factors observed in Cajun populations [2, 3]. Therefore, CRC prevention strategies targeting rural and underserved populations in Acadiana could mitigate CRC‐related geographic disparities in Louisiana.

Despite its prevalence, CRC is one of the most preventable and treatable cancer types, largely due to the effectiveness of screening methods for early detection. CRC screening has received a Grade A, the highest‐level recommendation, by the United States Preventive Services Task Force (USPSTF) for adults between 50 and 75 years old, and a Grade B for 45 to 49‐year‐olds [4]. Furthermore, an estimated 68% of CRC deaths could be prevented if all people were appropriately screened [5]. However, adherence to screening is only about 59% in the US [6] and even lower (~43%) among patients of Louisiana federally qualified health centers (FQHCs) [7], which provide care regardless of ability to pay for services. Moreover, rural populations experience increased barriers to screening, including high screening costs, lack of insurance coverage, and lack of awareness [8]. Therefore, there is a need to implement and evaluate evidence‐based strategies for improving the uptake of CRC screening at FQHCs serving rural and other at‐risk communities in Louisiana.

Meta‐analytic evidence suggests that patient navigation and mailed stool‐based screening kits are among the most effective interventions for increasing CRC screening [9], especially for populations experiencing health disparities [10, 11]. Evidence across colorectal, cervical, and breast cancer screening has found that adding patient financial incentives may increase completion in some circumstances [12, 13, 14, 15], but effectiveness data from FQHCs or other low‐income or at‐risk communities using community‐driven approaches are limited. Moreover, intervention effects vary based on incentive structure and other implementation strategies [12, 13, 14, 15], suggesting that tailoring approaches to the local community context could be key to success. Prior cancer screening interventions in underserved populations using community‐tailored feedback have been successful [16, 17, 18, 19, 20].

In the present study, we examined the feasibility and preliminary effectiveness of a financial incentive intervention for CRC screening at an FQHC serving patients in Acadiana. The intervention and study design were well informed by priorities and feedback from FQHC and other community partners in Louisiana. We hypothesized that (1) the intervention would demonstrate preliminary effectiveness for improving uptake of CRC screening and (2) the study design would demonstrate sufficient feasibility to support a larger follow‐up RCT, based on recruitment and retention outcomes.

## 2 Methods

### 2.1 Participants and Procedures

This randomized controlled trial (RCT) was conducted within clinical care delivery at an FQHC clinic in Acadiana from November 11, 2023 to March 31, 2024 (NCT06124131) as an initiative of the Louisiana Community Engagement Alliance (LA‐CEAL). The mission of LA‐CEAL is to address pressing public health challenges and health disparities in Louisiana by collaborating with community partners, including FQHCs, to disseminate, implement, and evaluate evidence‐based practices in Louisiana communities. Eligible participants were patients of the FQHC clinic who were (1) age 45–75; (2) due for CRC screening (i.e., had not had a colonoscopy in last 10 years, sigmoidoscopy or colonography in last 5 years, Cologuard screening in last 3 years, or other stool‐based test in last year); and (3) able to understand and speak English. Participants were excluded if they were currently participating in another clinical trial or research study on CRC screening or were unable or unwilling to give informed consent. Potentially eligible participants were identified through the electronic health record (EHR) by FQHC staff and were contacted via phone or in person at a clinic appointment. After confirming eligibility, patients provided informed consent before they were randomly assigned within REDCap to either the intervention or control group. Outcomes and key demographics, including race and ethnicity, were extracted from the EHR. This study was approved by the Tulane University Institutional Review Board.

### 2.2 Intervention

#### 2.2.1 Patient Outreach and Navigation

Participants in both groups received outreach reminders, education, and navigation about CRC screening, flu vaccine, and COVID‐19 vaccine during a phone call or in‐person discussion with a primary care staff member lasting about 10 min. The intervention was delivered by a single primary care staff member with experience as a clinical research coordinator for other community‐based studies, who was trained on the data collection and outreach call protocols. Outreach primarily focused on offering participants a stool‐based CRC screening kit, allowing the choice between two options: (1) Cologuard (completed once every 3 years, test kit mailed directly to participant's home from Cologuard with collected stool sample mailed back to Cologuard from participant) or (2) immunochemical fecal occult blood (iFOB) test (completed once every year, test kit can be mailed to participant's home or picked up at clinic, collected stool sample can be mailed back or dropped off at the clinic by participant). Either test was provided to patients by their primary care clinic free of charge; if the test is positive, a follow‐up colonoscopy is recommended. The intervention script was adapted from two Evidence‐Based Cancer Control Programs (EBCCP) [21, 22] for CRC screening [23], which provided telephone outreach about the benefits of CRC screening, addressing common questions and barriers to screening. In addition to providing information on CRC and screening, the intervention script included a table at the end with recommended responses to frequently asked questions (e.g., what is colorectal cancer? What are the screening guidelines?) and barriers to patient adherence to CRC screening (e.g., too busy, concerned about expense), which was based on previous patient navigation‐based EBCCPs [22]. After discussing CRC screening, the outreach script ended with a brief discussion about eligibility for and availability of the 2023–2024 seasonal flu and COVID‐19 vaccines.

#### 2.2.2 Financial Incentives for Intervention vs. Control

Participants in both groups received the outreach and navigation described above. Additionally, participants randomized to the intervention group received a $50 incentive per service, up to a total of $150, for completing each of the following services: CRC screening, flu vaccine, and COVID‐19 vaccine. Participants randomized to the control group received a $50 incentive per service, up to a total of $100, for completing their flu vaccine and COVID‐19 vaccine, but did not receive an incentive for completing CRC screening. This incentive amount was selected by considering community partner feedback, local research norms, and amounts offered by payers for completion of similar preventive services. Providing incentives to both groups for flu and COVID‐19 vaccines was recommended to allow all patients to receive incentives for participating, while also isolating the effect of CRC screening incentives.

### 2.3 Outcomes and Analysis

#### 2.3.1 Sample Size Determination

Based on recommendations for pilot and feasibility studies [24, 25, 26, 27], we determined that a sample size of 50 participants would be sufficient for identifying any unanticipated study design problems and for estimating the feasibility outcomes, while also minimizing community burden. This is consistent with prior pilot and feasibility trials of outreach interventions for cancer prevention [28, 29, 30, 31].

#### 2.3.2 Preliminary Effectiveness

The primary effectiveness outcome was CRC screening uptake, defined as EHR‐documented completion of CRC screening within 2 months post‐enrollment. Secondary effectiveness outcomes explored whether there was a difference between groups in EHR‐documented completion of the following services within 2 months post‐enrollment: (1) flu vaccine, (2) COVID‐19 vaccine, (3) any of the three services, and (4) all three services. Analyses were conducted in R version 4.3.1. Binary variables were created to indicate study group (intervention vs. control) and receipt of each outcome variable during the 2‐month follow‐up period. Assumptions of the chi‐squared test of independence to test the between‐group differences in each outcome were assessed [32]. Due to small cell sizes for some effectiveness outcomes, Fisher's exact tests were used [32].

#### 2.3.3 Feasibility

We documented the following metrics to evaluate the feasibility of implementing the intervention: number of patients who (1) were reachable by phone or in person, (2) began the eligibility screener, (3) consented and were randomized, and (4) completed the outreach call. The data collection form also included a checklist of common self‐reported barriers to CRC screening. If participants initially declined a stool‐based screening test, they were prompted to select the reason(s) from a checklist (e.g., too busy, financial concerns, don't know how to complete test, don't see need, or specifying “other” in free text).

## 3 Results

### 3.1 Sample Characteristics

Table 1 presents participant demographic characteristics at baseline: 56.0% White, 42.0% Black, and the average age was 52.4 years old. Most participants were female (70.0%), non‐Latina/o/X (98%), and were either uninsured (24.0%) or on Medicaid (50.0%). Among participants who had health insurance (*n* = 36 [72% of sample]), none were aware of a financial incentive offered by their health insurance provider for completing CRC screening. A small proportion of participants had already received their seasonal flu vaccine (8.0%) or COVID‐19 vaccine (2.0%) at study enrollment.

**TABLE 1:** Baseline participant characteristics.

Characteristic	Total (*N* = 50)	Intervention (*n* = 25)	Control (*n* = 25)
Age, mean (SD)	52.40 (6.61)	52.48 (6.63)	52.32 (6.74)
Female sex, *N* (%)	35 (70.0%)	15 (60.0%)	20 (80.0%)
Race, *N* (%)			
White	28 (56.0%)	14 (56.0%)	14 (56.0%)
Black	21 (42.0%)	10 (40.0%)	11 (44.0%)
Other	1 (2.0%)	1 (4.0%)	0 (0.0%)
Latina/o/x, *N* (%)	1 (2.0%)	1 (4.0%)	0 (0.0%)
Health insurance, *N* (%)			
Commercial/private	4 (8.0%)	3 (12.0%)	1 (4.0%)
Medicaid	25 (50.0%)	10 (40.0%)	15 (60.0%)
Medicare	7 (14.0%)	4 (16.0%)	3 (12.0%)
Uninsured	12 (24.0%)	7 (28.0%)	5 (20.0%)
Unknown/missing	2 (4.0%)	1 (4.0%)	1 (4.0%)
Previously vaccinated for flu (2023–2024 season), *N* (%)	4 (8.0%)	2 (8.0%)	2 (8.0%)
Previously vaccinated for COVID‐19 (2023–2024 season), *N* (%)	1 (2.0%)	1 (4.0%)	0 (0.0%)

### 3.2 Preliminary Effectiveness

Completion of CRC screening (primary effectiveness outcome) from baseline to 2‐months post‐enrollment was significantly higher in the intervention versus control group (*n* = 17 [68%] vs. *n* = 8 [32%], respectively, *p* = 0.02) (Table 2). For secondary outcomes, there were no statistically significant differences between groups in the uptake of the flu vaccine, COVID‐19 vaccine, or a composite measure of all three services from baseline to 2‐months post‐enrollment. There was a trend for higher completion of any service in the intervention versus control group (80% vs. 52%, *p* = 0.07), with CRC screening being the most received service.

**TABLE 2:** Between‐group differences in primary and secondary study outcomes, *n* (%).

Outcome	Group	Baseline	2 months	Completion within 2 months post‐enrollment	*p*
Primary study outcome					
Colorectal cancer (CRC) screening	Intervention	0 (0%)	17 (68%)	17 (68%)	0.02
	Control	0 (0%)	8 (32%)	8 (32%)	
Secondary study outcomes					
Flu vaccine	Intervention	2 (8%)	11 (44%)	9 (36%)	> 0.99
	Control	2 (8%)	12 (48%)	10 (40%)	
COVID‐19 vaccine	Intervention	1 (4%)	2 (8%)	1 (4%)	> 0.99
	Control	0 (0%)	0 (0%)	0 (0%)	
At least 1 service^a^	Intervention	2 (8%)	20 (80%)	20 (80%)	0.07
	Control	2 (8%)	15 (60%)	13 (52%)	
All 3 services^b^	Intervention	0 (0%)	2 (8%)	1 (4%)	> 0.99
	Control	0 (0%)	0 (0%)	0 (0%)	

*Note: N* = 50 (*n* = 25 [control group], *n* = 25 [intervention group]). *p*‐values obtained from Fisher's exact tests comparing between‐group differences in the proportion of participants who completed each outcome within 2 months post‐enrollment. The intervention group received $50/service for CRC screening, flu vaccine, and COVID‐19 vaccine. The control group received $50/service for flu vaccine and COVID‐19 vaccine, but no incentive for CRC screening.

^a^ CRC screening, flu vaccine, or COVID‐19 vaccine. Presence at baseline and completion within 2 months post‐enrollment are not mutually exclusive (e.g., an individual could have at least one service already present at baseline and still complete at least one other service between baseline and 2 months).

^b^ CRC screening, flu vaccine, and COVID‐19 vaccine. Since participants who had a flu or COVID‐19 vaccine present at baseline were still included in the trial, not all participants had to complete all three services between baseline and post‐enrollment to have all three services present at 2 months.

### 3.3 Feasibility

**FIGURE 1:**
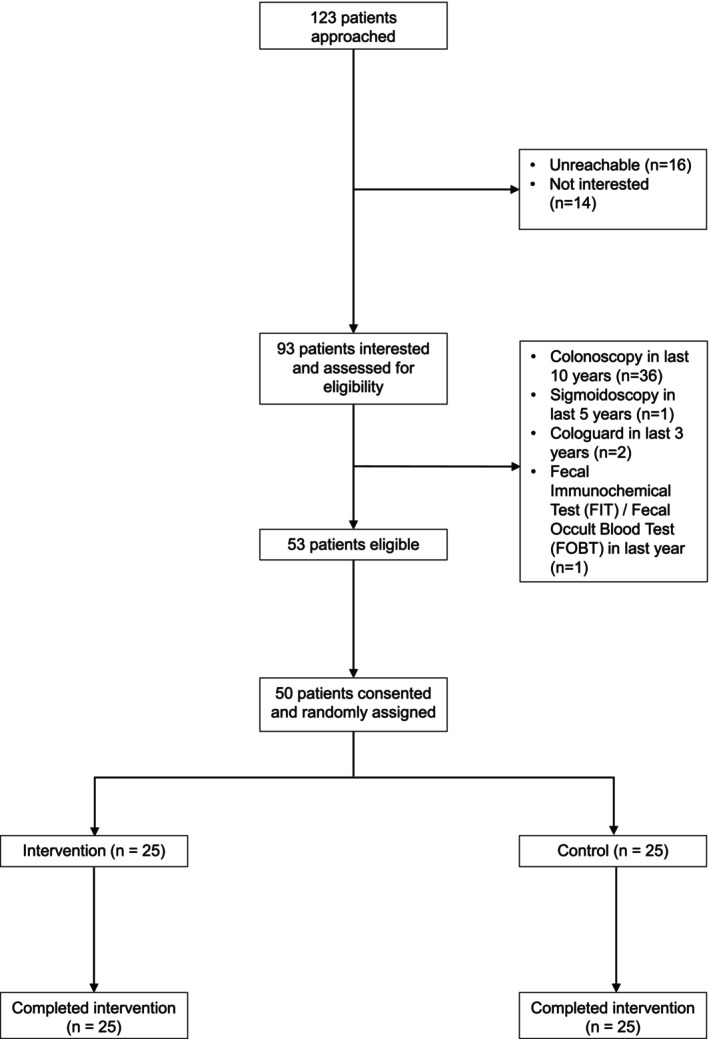
Recruitment and randomization from November 11, 2023 to January 24, 2024.

From November 11, 2023 to January 24, 2024, 123 potentially eligible patients were approached to participate in the study (Figure 1). One hundred and seven patients (87.0%) were reachable, 93 (75.6%) were interested in participating and underwent assessment for eligibility, and 53 were eligible to participate (57.0% of screened). Of 53 eligible participants, 50 (94.3%) consented to participate and underwent randomization, and 100% of those randomized completed the intervention outreach call or conversation. All participants initially agreed to receive a stool‐based CRC test during the call, thus no barriers to uptake of the CRC screening test were reported. Compared to enrolled participants, the 73 other approached individuals were 3 years older on average (55.50 vs. 52.40, *p* = 0.02), but comparable in terms of sex (69.9% female), race (46.6% Black), and insurance (61.6% Medicaid/uninsured), *p* > 0.12 for each comparison.

## 4 Discussion

A pilot study of a financial incentive intervention for CRC screening was feasible and effective for increasing CRC utilization within a primary care setting in Acadiana, Louisiana. The intervention group, which received financial incentives for CRC screening, was more than twice as likely to complete CRC screening within 2 months post‐enrollment compared to a control group. Moreover, > 50% of interested individuals were eligible to participate and > 90% of eligible individuals enrolled in the study, demonstrating that the study design was feasible. These results are encouraging and call for a follow‐up trial in a larger sample to confirm effectiveness.

These findings contribute to knowledge on how to successfully design and implement patient financial incentive interventions for CRC screening for primary care settings in low‐income and underserved communities. While past research found that patient financial incentives may provide only a modest benefit for cancer screening uptake [12, 13, 14, 15], our intervention was uniquely informed by community feedback, which has been shown to improve the effectiveness of cancer prevention outreach programs [16, 17, 18, 19, 20]. For instance, previous studies provided patients with a fixed incentive of $5–$20 [12], but our community partners provided insight that $50 may be more incentivizing for their patient population and more consistent with other statewide incentive programs such as those provided by Louisiana Medicaid Health Plans. Moreover, stakeholders suggested that incentives for flu and COVID‐19 vaccines be offered to both groups alongside the CRC incentives for the intervention group to improve acceptability. Our findings suggest that since the partial incentive (control) group and the full incentive (intervention) group did not differ on likelihood to receive flu or COVID‐19 vaccines, lacking incentives for some services may not deter patients from taking advantage of other incentivized services. Additionally, the intervention was delivered by FQHC staff and integrated into the clinic's routine outreach for CRC screening; emphasis on community and clinic engagement may have contributed to the success of the intervention.

If found effective in larger follow‐up studies, the intervention could have implications for improving FQHC healthcare delivery and increasing CRC screening rates in Louisiana by targeting an underserved and at‐risk population [2, 3]. Since CRC screening is a quality indicator for enhanced reimbursement [33], ensuring performance on this measure is also critical for facilitating overall access to healthcare among low‐income and uninsured/underinsured populations. Many health insurance payers, including UnitedHealthcare, Cigna, and some Louisiana Medicaid managed care organizations, provide varying financial incentives to their members for completing preventive health measures such as CRC screening. While a systematic review found that interventions addressing social determinants of health barriers to cancer screening, including patient financial incentives, are cost‐effective [34], additional cost‐effectiveness studies in CRC screening are needed to identify the optimal incentive amount and frequency. Finally, since this study found that no participants were aware of any available incentives from their health insurance provider, incentive programs may be more effective if delivered through participants' trusted healthcare clinics or may need to be coupled with outreach by healthcare staff in primary care clinics to raise awareness about the programs.

### 4.1 Limitations and Strengths

The study findings should be considered in light of limitations. Foremost, the sample size was limited to 50 participants and may not be generalizable. While the proportion of Black and female participants was comparable to the clinic's population, only 2% were Latino/a/x (vs. up to 10% of the clinic's population) [7]. Follow‐up studies with larger and more representative samples should be conducted to confirm findings and evaluate effectiveness across demographics. Furthermore, similar work in patient populations with more men is needed. Nevertheless, the study collected important feasibility data for evaluating the intervention in an understudied high‐risk population. Second, while stakeholder engagement facilitated the study's effectiveness in the local context, this tailored approach may contribute to a lack of generalizability to other populations or settings. Future research should continue to employ community‐engaged strategies to understand local effectiveness. Third, although we collected data on barriers to receiving the CRC screening test for those who declined the test, we did not collect data on barriers to using the test among those who agreed to the test, which should be explored in future work. Finally, due to reliance on the local clinic's EHR as the primary data source, it is possible that the CRC completion rate was underreported in this study if some participants completed the screening at a different institution, and the collection of demographic characteristics was relatively limited. However, this pragmatic design also reduced the burden on participants and clinical staff implementing the study, while still allowing the collection of data on intervention effectiveness.

This study found that financial incentives may be an effective strategy to increase CRC screening completion in a primary care setting in Acadiana. Future research should confirm findings in larger samples and explore implications for health outcomes including CRC diagnosis, mortality, and quality of life.

## Conflicts of Interest

The authors declare no conflicts of interest.
